# Massage in the Treatment of Muscular Rheumatism

**Published:** 1897-02

**Authors:** 


### MASSAGE IN THE TREATMENT OF MUSCULAR RHEUMATISM.

Graham, (*American Journal Medical Science*, Vol. ÇVI., No. 2, *Modern Medicine*,) points out that lumbago may arise from a cold, strain, fatigue or rheumatism. Its pathology is probably coagulation of the semi-fluid contractile muscular substance, and adhesion of muscular fibrils, so that motion gives rise to partial, irregular and painful contractions. Recent cases of muscular rheumatism are almost invariably cured by massage. The author further states that the same result may be brought about, though not so rapidly, by rest, warmth, electricity, or the use of salicylate of soda. Five different actions are claimed for massage by the author, namely, mechanical, thermal, electrical, nervous and chemical, and he suggests that when a case of apparently muscular rheumatism does not only not yield, but does not remain improved after massage, the probability is that the case is one of neuritis, affecting the nerve fibers that supply the impaired muscles. This probability will be strengthened if the pain is uniform, affecting the same muscles on both sides, if it is worse at night, when at rest and warm in bed, and better when up and moving about. In the case of muscular rheumatism it would be aggravated by motion, and relieved by rest and warmth. Massage may thus be used as a means of diagnosis between muscular rheumatism and neuritis.—*N. Y. Med. Times.*

---

### SALOL DANGEROUS IN NEPHRITIS.

Dr. James Tyson reports two cases of nephritis in which the administration of salol in combination with bismuth to check a too free diarrhœa was followed by suppression of urine—in one case causing death, in the other very serious symptoms. He therefore considers it advisable to warn the profession of the possible danger of administering this drug for any purpose to patients suffering from nephritis.

---

### TO REMOVE POWDER STAINS.

EDITOR *Medical World:* The best treatment to get rid of powder imbedded in the skin is to apply a solution of corrosive sublimate, gr. vi. to f. oz. of water, by means of a quill to the entrance of each grain, which causes suppuration around the grain of powder, and by wiping with a cloth all the grains will be brought out and no stain will remain.

W. D. HARTMAN, M. D.

*West Chester, Pa.*

